# Supplementary material for: Investigating Antibody Reactivity to the Intestinal Microbiome in Severe Myalgic Encephalomyelitis/Chronic Fatigue Syndrome (ME/CFS): A Feasibility Study
Source: Int J Mol Sci. 2023 Oct 18;24(20):15316. doi: 10.3390/ijms242015316 (PMC10607161; doi:10.3390/ijms242015316)
Supplement: Supplementary file 1 [file ijms-24-15316-s001.zip › ijms-2635016-SI.pdf]

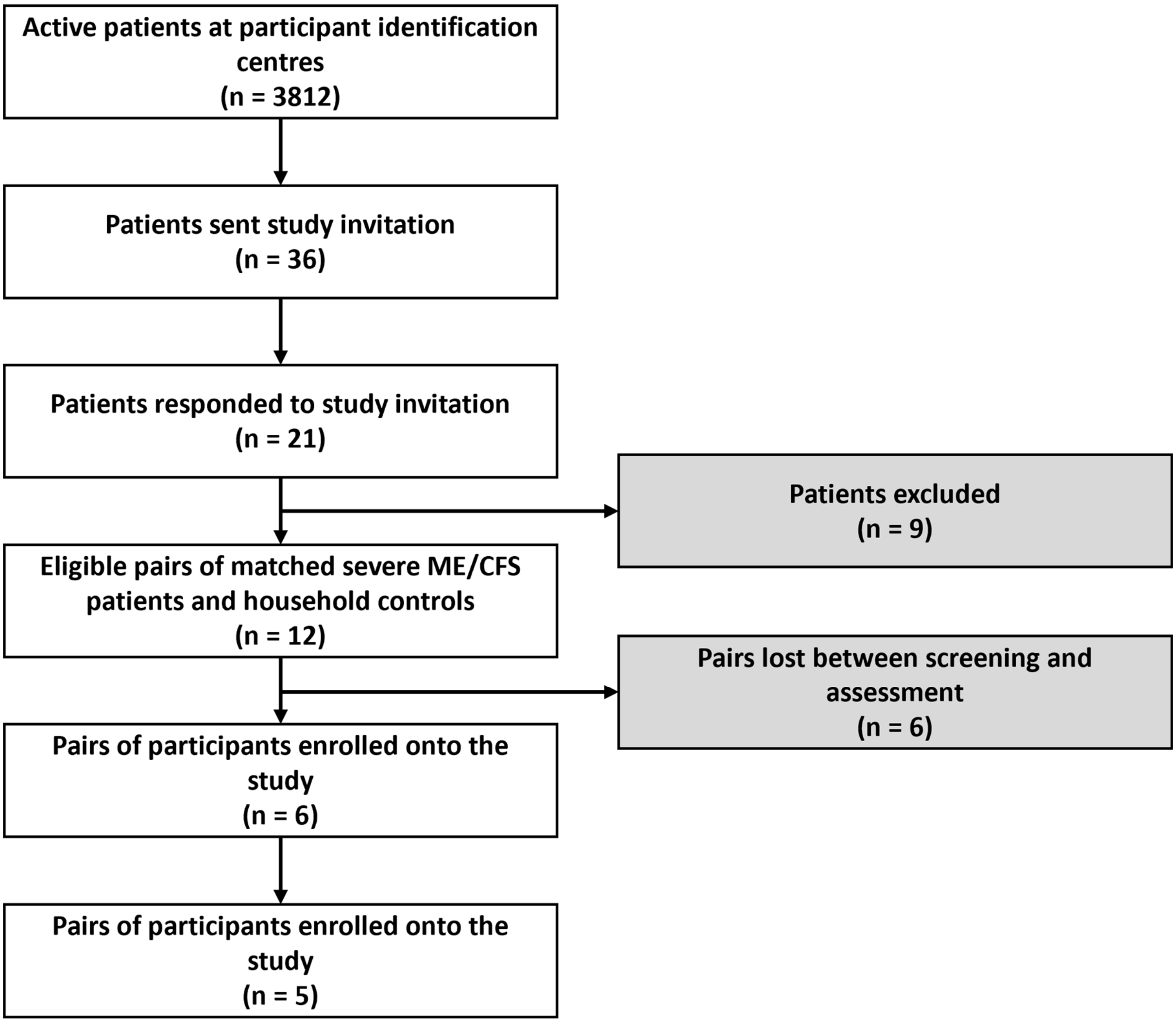

Figure S1. Study participant recruitment pathway. Severe ME patients were recruited from the CFS clinic at Epsom and St Helier University Hospitals (ESTH), Carshalton, UK and the ME/CFS service at East Coast Community Healthcare Centre, Lowestoft, UK.

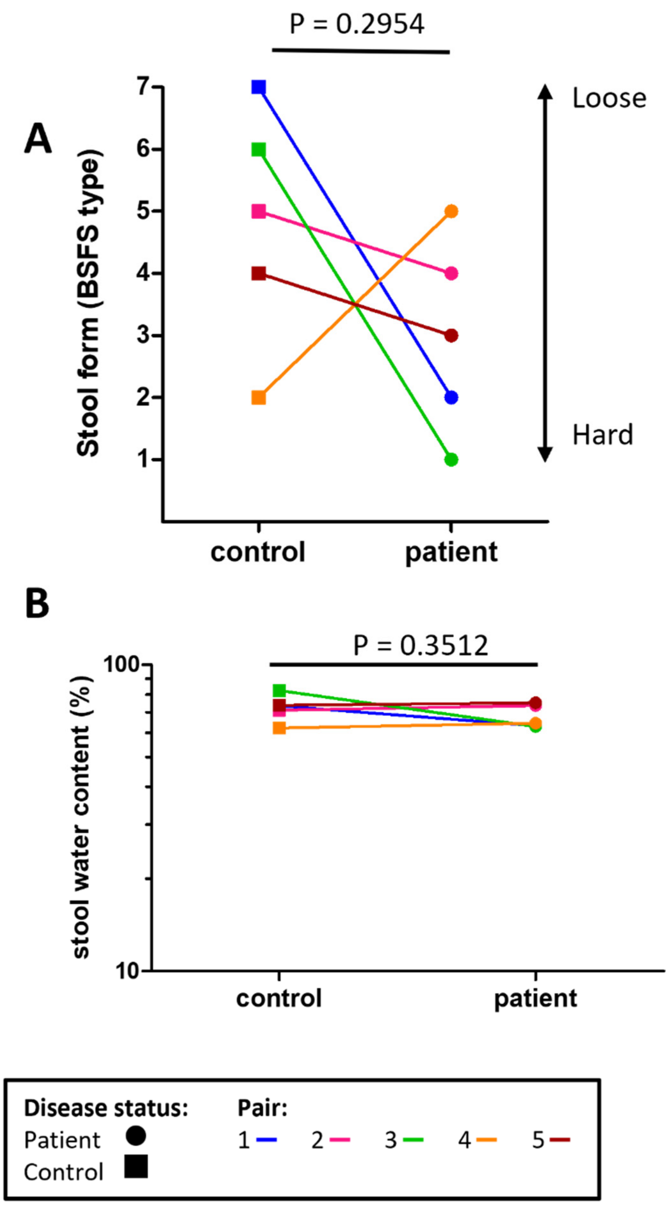

Figure S2. Stool consistency: (**A**) Analysis of stool consistency using the Bristol stool form scale (BSFS) in severe ME/CFS patients (n=5) and matched household controls (n=5); (**B**) Water content in stool samples in severe ME/CFS patients (n=5) and matched household controls (n=5). *P values* were calculated using a two-tailed paired *t*-test.

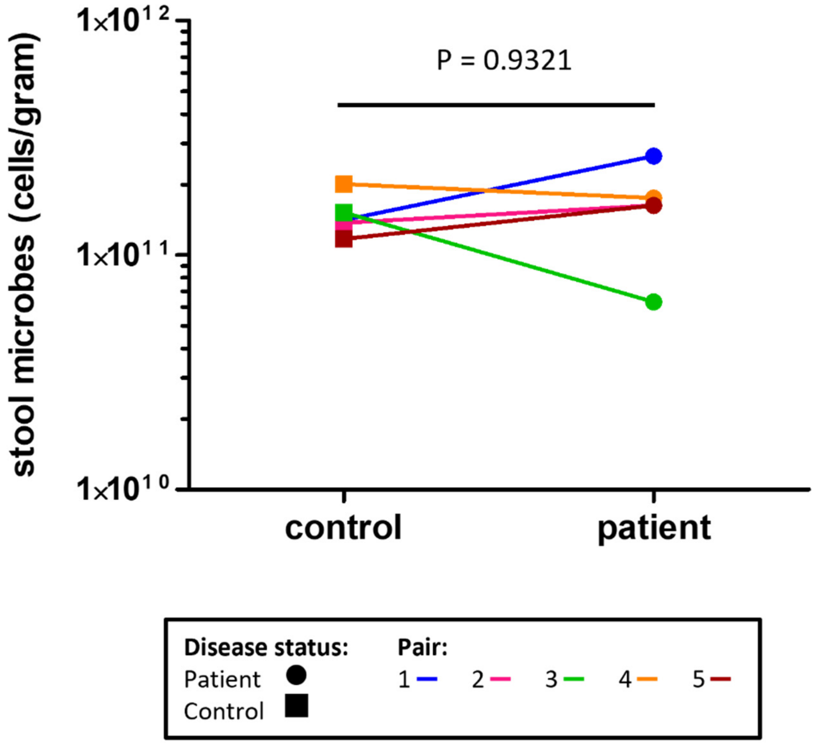

Figure S3. Stool microbial load. Flow cytometric analysis of SYBR Green+ microbial load in stool samples of severe ME/CFS patients (n=5) and matched household controls (n=5).

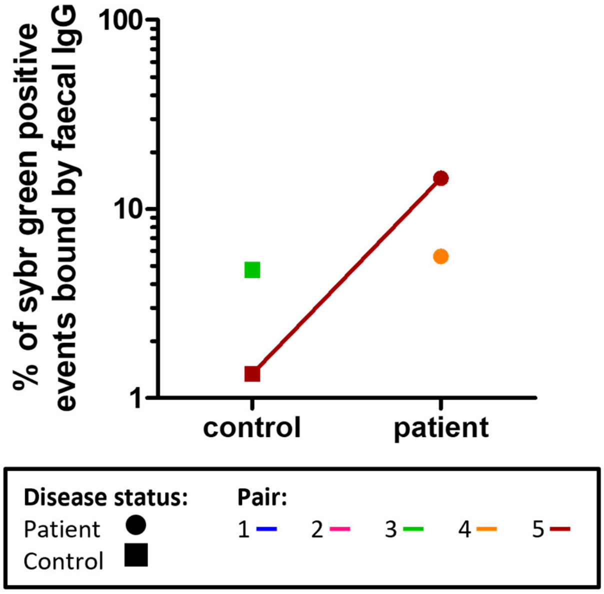

Figure S4. Proportion of stool microbes bound by faecal IgG. Flow cytometric analysis of SYBR Green+ IgG+ microbes in stool samples of severe ME/CFS patients (n=5) and matched household controls (n=5).

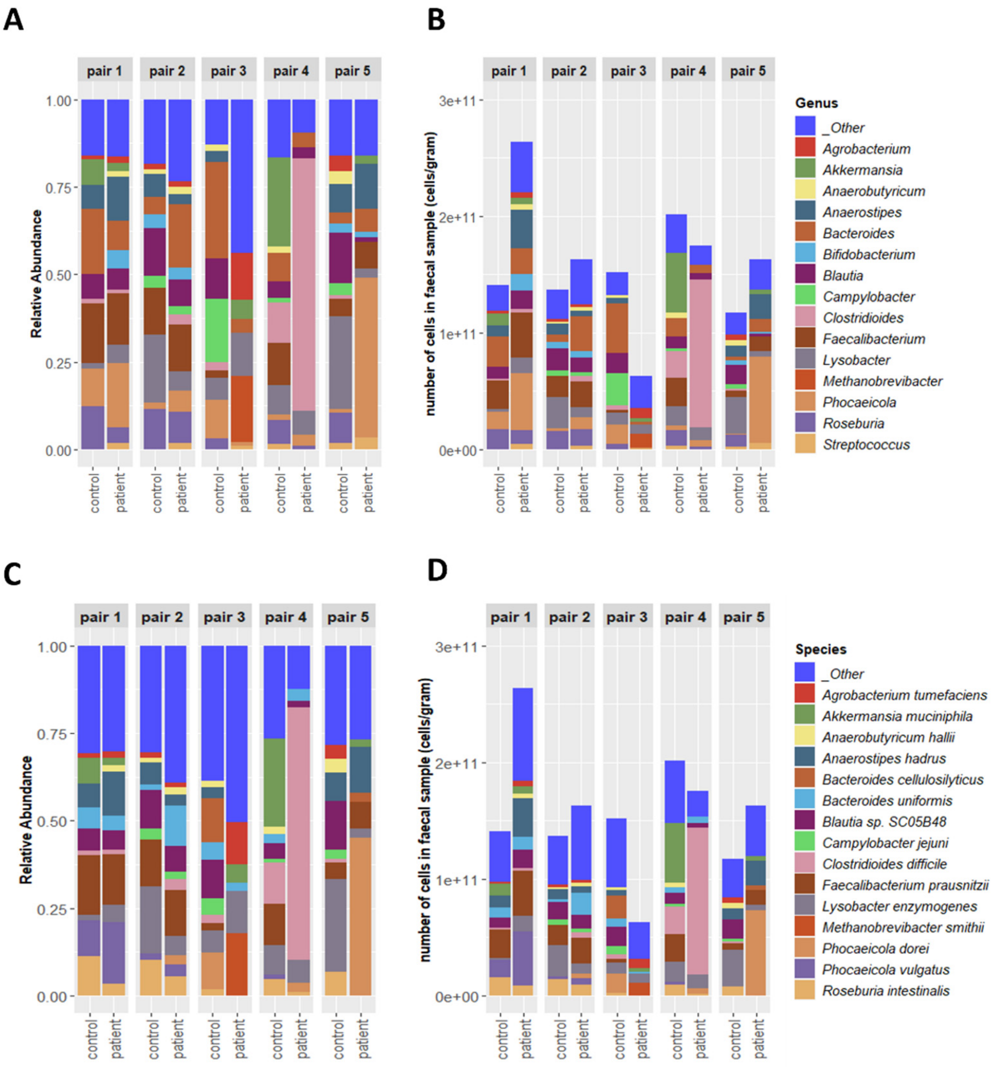

Figure S5. Stool microbiome profiling of severe ME/CFS patients (n=5) and matched household controls (n=5): (**A**) Relative microbiome profiling (RMP) at the genus-level; (**B**) quantitative microbiome profiling (QMP, cells per gram of faeces) at the genus-level; (**C**) RMP at the species-level, (**D**) QMP at the species-level.

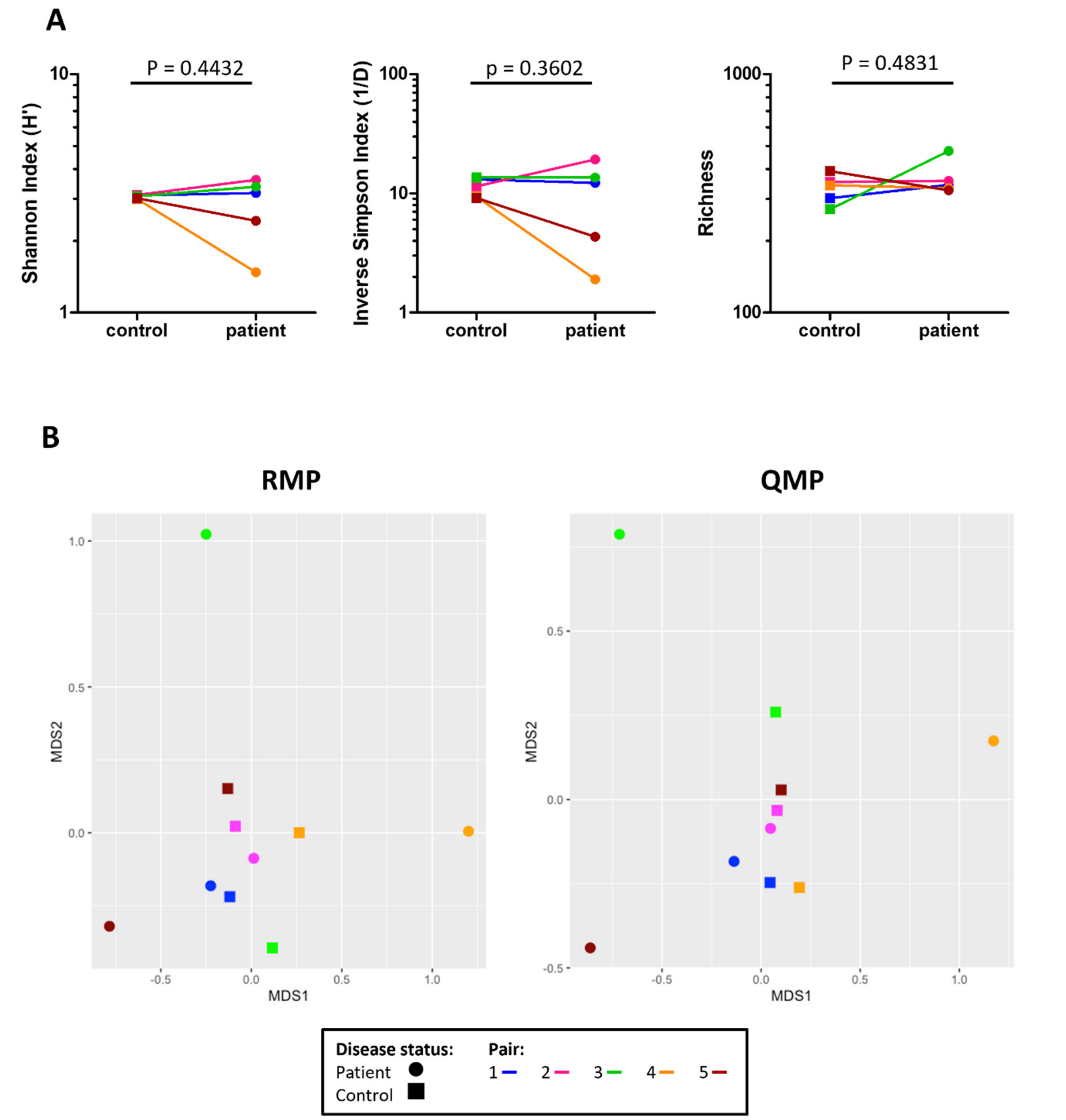

Figure S6. Pairwise alpha- and beta-diversity comparisons of the stool microbiomes of severe ME/CFS patients (n=5) and matched household controls (n=5). Analyses were performed at the species-level on shotgun metagenomics data from SYBR+ stool microbes; (**A**) Alpha diversity measures of Shannon index, inverse Simpson index and richness based on reads rarefied to the lowest sequencing depth; (**B**) Beta diversity of relative microbiome profiling (RMP) and quantitative microbiome profiling (QMP, cells per gram faeces). Beta-diversity was calculated using Bray-Curtis dissimilarity, presented on a non-metric multi-dimensional scaling (NMDS) plot. *P* values were measured using two-tailed paired *t*-tests.

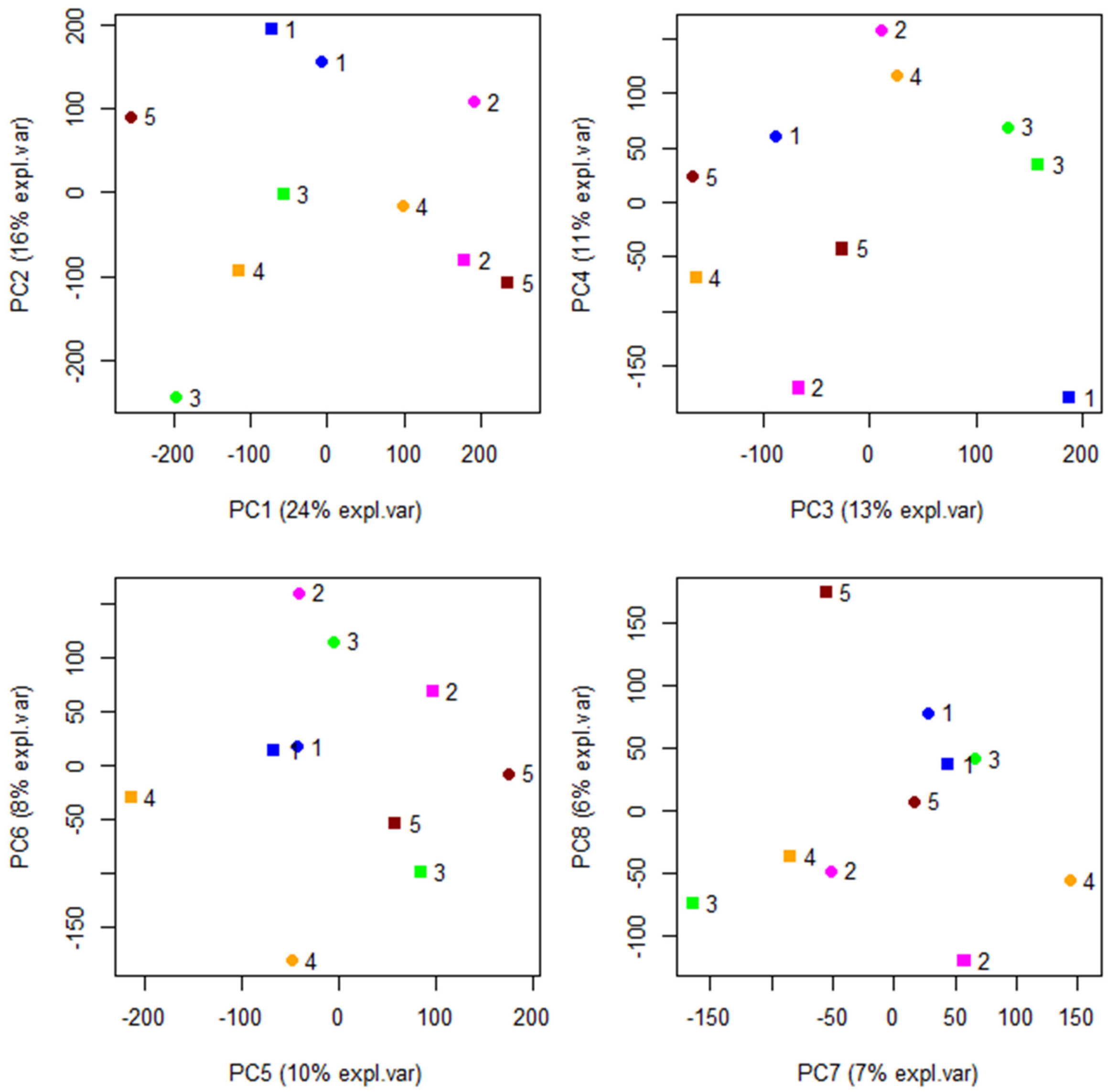

Figure S7. Functional composition of stool metagenomes from severe ME/CFS patients (circles) (n=5) and matched household controls (squares) (n=5). Principal component analysis (PCA) of the relative abundances of gene families. Pair numbers are depicted on the graph but were not used in analysis.

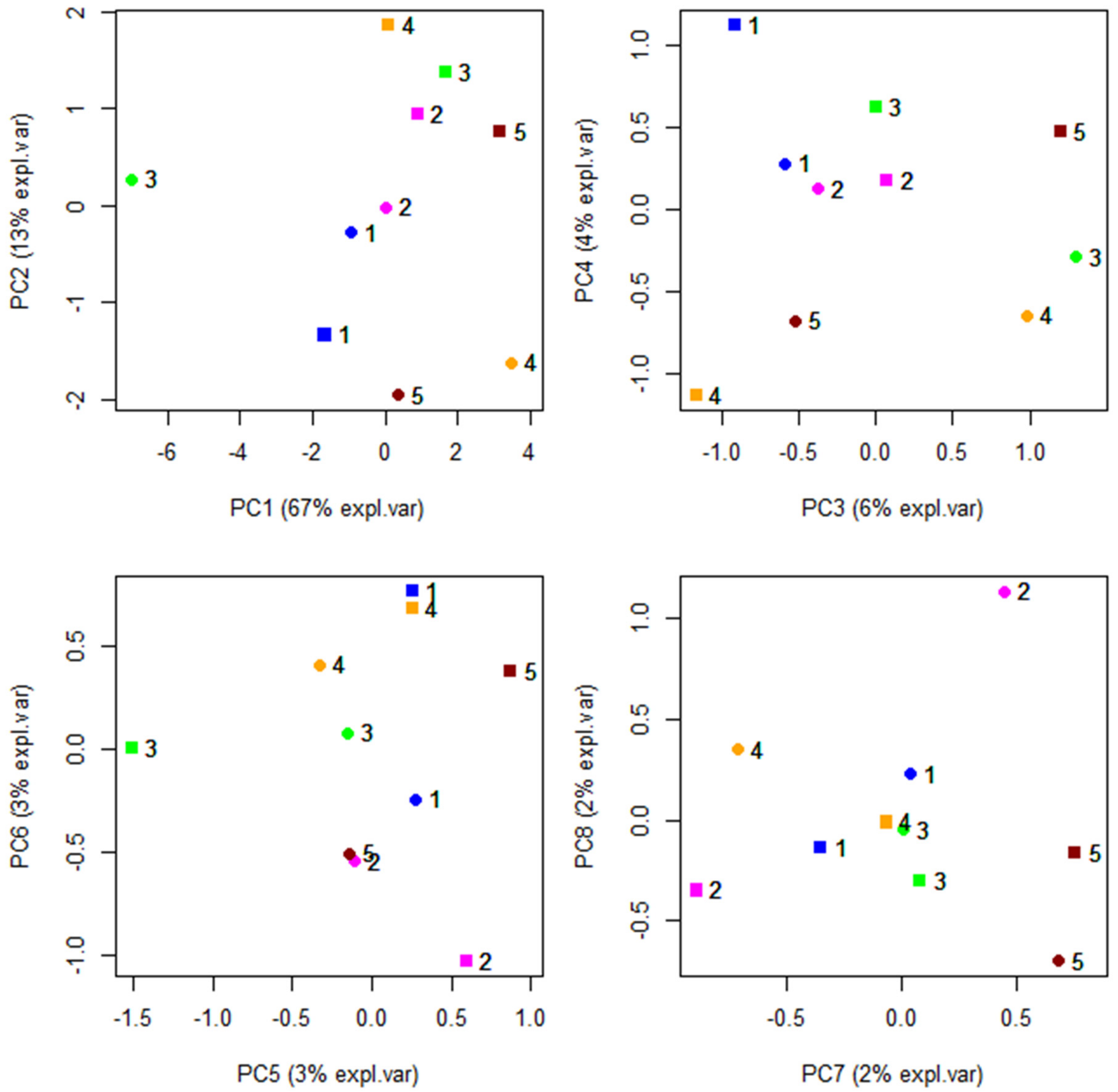

Figure S8. Functional profiling of stool microbes reactive with serum IgG. Principal component analysis (PCA) of IgG probability ratios of gene families from the stool microbiome in severe ME/CFS patients (circles) (n=5) and matched household controls (squares) (n=5). Pair numbers are depicted on the graphs but were not used in analysis.

Table S1. The size of IgG positive and IgG negative fractions collected during IgG-Seq. This data was used to calculation of IgG probability ratio scores.

Pair	Participant	Fraction Sizes (%)	
		IgG+	IgG−
1	Patient	21.1	79.5
	Control	18.0	79.8
2	Patient	35.1	58.1
	Control	48.1	49.7
3	Patient	17.9	65.5
	Control	46.3	44.9
4	Patient	65.0	29.0
	Control	33.7	63.1
5	Patient	24.2	75.3
	Control	62.7	31.1
